# Laparoscopic vaginal bead pull-through vaginoplasty technique using dental prosthesis material

**DOI:** 10.4274/tjod.galenos.2020.39112

**Published:** 2020-10-02

**Authors:** Çetin Kılıççı, İlhan Şanverdi, Ezgi Darıcı, Enis Özkaya

**Affiliations:** 1 University of Health Sciences Turkey, Zeynep Kamil Women and Children’s Diseases Training and Research Hospital, Clinic of Obstetrics and Gynecology, İstanbul, Turkey

**Keywords:** Absent vagina, Mayer-Rokitansky-Küster-Hauser, primary amenorrhea, vaginoplasty

## Abstract

**Objective:**

Many reconstructive surgical procedures have been described for vaginal agenesis. Almost all are surgically challenging, multistage, time-consuming or leave permanent scars on the abdomen or skin removal areas. The aim of this study was to introduce a simple and cheaper approach for laparoscopic vaginal bead-pull through.

**Materials and Methods:**

In this retrospective study, we report a total of six patients with congenital absence of vagina [Mayer-Rokitansky-Küster-Hauser (MRKH) syndrome] who were treated with a laparoscopic vaginal bead pull-through technique between 2018 till 2019 with a dental prosthesis material.

**Results:**

Six patients with MRKH syndrome were treated with a laparoscopic vaginal bead pull-through technique. None of the women had any previous treatment. The mean age at the time of surgery was 18.7±3.1 years and mean body mass index was 25 (range, 19-38) kg/m^2^. None of the patients had any additional malformations. In all patients, normal external genitalia and complete vaginal agenesis were observed during examination. The mean duration of surgery was 72 (range, 55-95) minutes. All patients were discharged on the 3^rd^ postoperative day. No intraoperative complications were encountered. All patients had their urinary catheters removed within 12 hours after surgery. The mean vaginal length at discharge was 10 (range, 8-13) cm and all patients had adequate vaginal diameter, allowing introduction of three fingers easily. At the 12^th^ postoperative month, the mean vaginal length was 9.2±1.6 cm. All patients had complete epithelization. All the women were sexually active one year after surgery.

**Conclusion:**

The laparoscopic vaginal bead pull-through technique using dental prosthesis material can provide satisfactory results with shorter surgical time and lower cost.

**PRECIS:** The laparoscopic vaginal bead pull through technique using dental prosthetic material can provide satisfactory results with shorter surgical time and lower cost.

## Introduction

Development of the mullerian canal is one of the most incomprehensible issues in gynecology. Each section of the mullerian canal has different reproductive functions. Complete absence of mullerian development leads to aplasia; the common form of partial development leads to tubal and partial uterine development, and complete absence of the upper three-quarters of the vagina. In most cases of upper vaginal absence, the uterus is usually hypoplastic or primitive. The ovaries are normal, but are placed on the lateral pelvic wall along with the uterus. Classically, this is defined as Mayer-Rokitansky-Küster-Hauser (MRKH) syndrome. The estimated prevalence is about 1:4000 to 5000 women^(1)^. There are many surgical options to create a neo-vagina. Free skin graft^(2)^, intestinal or sigmoid vaginoplasty^(3)^, amniotic graft^(4)^, and pelvic peritoneum graft^(5)^ have been used for these procedures. The disadvantages of previously defined procedures were stenosis, poor lubrication, scarring, contracture leading to dyspareunia, and the need for laparotomy. Transformation from free skin graft to squamous cell carcinoma and sigmoid to adenocarcinoma has been reported^(6)^. Recently, there have been reports of neovagina formation with endoscopic help based on the Vecchietti technique^(7)^. The use of peritoneum in vaginoplasty was first described in the Russian literature. This method was made popular by Davydov^(8)^. The formation of a neovagina using a laparoscope was first described by Semm^(9)^. More recently reported techniques describe laparoscopic application to replace the original Davydov procedure.

The aim of this report was to introduce a technique using a laparoscopic vaginal bead pull-through technique using dental prosthesis material.

## Materials and Methods

After obtaining institutional ethics committee approval (University of Health Sciences Turkey, Zeynep Kamil Women and Children’s Diseases Training and Research Hospital 2020/36), in this retrospective study, six patients with MRKH syndrome were treated with a laparoscopic vaginal bead pull-through technique with dental prosthesis material from 2018 till 2019. The patients were followed from postoperative day 7 to a maximum of 12 months. The patients’ ages ranged between 15 and 24 years. Apart from the routine preoperative study, a diagnostic laparoscopy was performed to see the size and position of the uterus in the lateral pelvic walls to determine the feasibility of creating a pelvic anatomy to create a route to pull through the bead and to determine the possibility of neovagina formation.

### Procedure

**Figure 1:**
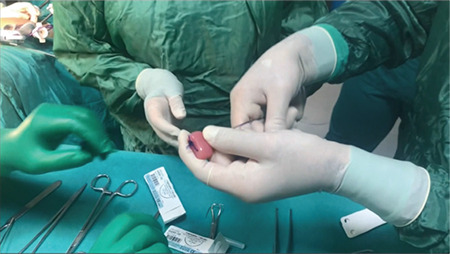
Cylindrical mass with two holes made of acrylic material used as a prosthesis in dentistry

**Figure 2:**
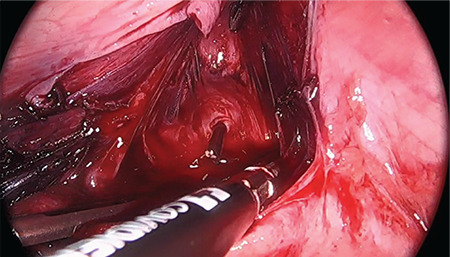
Pelvic peritoneal access using a Hegar cervical dilator through the neovagina

**Figure 3:**
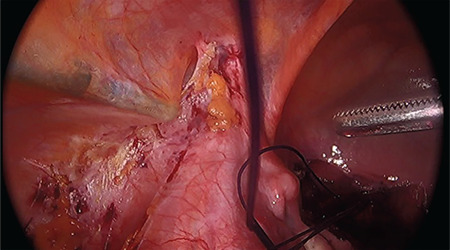
Extraperitoneally directed forceps to pull sutures from the neovagina to the lateral port sites

A modified vaginal bead set was prepared from an acrylic material that has been used as a prosthesis in dentistry (Figure 1). A vaginal bead was created to draw the blind vagina, which is 2 cm long, 1 cm wide, 1 cm high, with two holes in it. In order to apply internal traction to the bead, two polydioxanone (PDS) sutures were passed through the holes and their proximal ends were connected to each other under the vaginal bead. Following the modified set preparation, the abdomen was entered with a 10-mm trocar. Pneumoperitoneum was provided and suprapubic 5 mm trocars were placed on both sides. The bladder was removed cranioventrally from the anterior face of both round ligaments. The vaginal apex was sifted through the blind vagina using a thin Hegar cervical dilator, and the locations where the sutures were transported to the abdomen during laparoscopy were determined (Figure 2). The forceps of the 5 mm trocar were moved from under the peritoneum on both sides and the distal ends of the PDS sutures on the blind vaginal cuff were held with forceps and removed out of the abdomen under the peritoneum (Figure 3). Following abdominal washing, the procedure was terminated. The 10 mm trocar site was closed. The distal ends of both PDS sutures were pulled and traction was achieved with the vaginal bead in the blind vagina and the distal of the sutures were tied on a pad placed on the umbilicus. This pad on the umbilicus was used to stretch sutures to obtain the required vaginal length. The duration for hospital stay is 3 days in each case. After the traction technique is taught to the patient, she is discharged. Patients perform traction on their own at home, using oral analgesics. The prosthesis is removed after a total of one week. Following removal of the prosthesis, dilatation was continued with a suitable mold accompanied by local estriol cream for 2 weeks. If the patient has a partner, coitus is recommended two days a week. A lubricant with ginseng or hyaluronic acid is recommended during intercourse. All participants were reevaluated at 12^th^ months postoperatively to determine the vaginal length and sexual activity.

### Statistical Analysis

The statistical parameters were computed using the Statistical Package for the Social Sciences version 21.0 (SPSS Inc., Chicago, IL, USA). The continuous variables were expressed as the mean ± standard deviation. The European categorical variables were expressed as the number and percentage.

## Results

Six patients with congenital absence of vagina (MRKH syndrome) were treated using a laparoscopic vaginal bead pull-through technique.

None of the women had any previous treatment. The mean age at the time of surgery was 18.7±3.1 years and mean body mass index was 25 (range, 19-38) kg/m^2^. None of the patients had any additional malformations. In all patients, normal external genitalia and complete vaginal agenesis were observed during examination. The mean duration of surgery was 72 (range, 55-95) minutes. All patients were discharged on the 3^rd^ postoperative day. No intraoperative complications were encountered. All patients had their urinary catheters removed within 12 hours after surgery. The mean vaginal length at discharge was 10 (range, 8-13) cm and all patients had adequate vaginal diameter, allowing the introduction of three fingers easily. At the 12^th^ postoperative month, the mean vaginal length was 9.2±1.6 cm. All patients had complete epithelization. All women were sexually active one year after surgery.

## Discussion

In this report, we tried to present our case series of six women with congenital vaginal agenesis who underwent vaginoplasty with a bead pull-through technique using a dental prosthesis; our data analysis showed satisfactory results with lower cost.

MRKHS is caused by the hypoplastic embryologic development of the mullerian canal with the absence of the vagina, uterus or both^(10)^. Most patients have complete mullerian agenesis, and 47-84% of cases have uterine remnants with or without cavity^(11)^. As a result of anatomic insufficiency, patients are compromised in terms of sexuality and reproductive health. The main basis of MRKHS management is to create a new anatomically sufficient and satisfactory vagina^(12)^ to provide comfortable intercourse with minimal intervention. To allow this function, the new vaginal canal must meet the following conditions; secretory function for sufficient width, length, axis, and also lubrication. None of the many techniques^(13)^ proposed to date meet all these criteria. Following some conventional approaches for neovagina surgical techniques, newer modified forms of more satisfactory minimal invasive techniques have been introduced^(14)^; however, each procedure has been suggested to have specific disadvantages and complications based on the characteristics of the procedure and materials or tissues used to create a neovagina. A previously published review addressed all these specifically observed complications related to intestine, skin, buccal mucosa, and peritoneum^(15)^. Disadvantages specific for the procedures using peritoneum were defined as these procedures typically reserved for patients who have not had prior pelvic surgery and therefore its applications are limited. The risks of this procedure include injury to the bowel and bladder, as well as prolapse. Up to 23% of patients with a Davydov procedure will experience granulation tissue and 12% will have obliteration of the vaginal canal; furthermore, postoperative dilation is essential^(16)^. Self-lubricating neovagina has been provided by vaginal reconstruction using isolated bowel segments with low rates of failure and revision, additionally routine dilatation is not required for this procedure. It was reported that vaginoplasty using bowel was a safe and effective procedure^(17)^. Vecchietti and Davydov’s methods have been introduced as two commonly preferred laparoscopic options. The Vecchietti operation relies on passive upwards traction with an externally replaced spherical device rather than dilatation^(18)^, pain due to continuous traction and the need for prolonged hospitalization for continuous strong analgesia have been reported as the main disadvantages^(19)^. Finally, the device used for the technique has not yet been approved by the Unites States Food and Drug Administration, and it significantly increases the cost of the operation^(14)^. For this reason, some other alternative materials have been proposed to be used for this purpose^(20)^. In our proposal, we used a cheaper material that has been used as a prosthesis in dentistry, which indicates its safety for this procedure. The laparoscopic Davydov procedure is based on pulling down parietal peritoneum and suturing it to the vaginal introitus. A comparison of the procedures reveals that both Vecchietti and Davydov’s laparoscopic techniques are simple, safe, and effective surgical methods for vaginal reconstruction; Vecchietti’s procedure is more time-efficient and minimally invasive, on the other hand, Davydov’s procedure is associated with less pain, a longer vagina, and greater sexual satisfaction^(11)^. Evidence shows that laparoscopy-assisted peritoneal vaginoplasty by pushing down the peritoneum offers the advantages of reduced costs, complications, hospitalization, surgical time, and pain over the traditional technique^(21)^; however, further modifications may provide additional advantages for these approaches.

## Conclusion

The laparoscopic bead pull-through technique using dental prosthesis material can provide satisfactory results with shorter surgical time and lower cost. As the dental prosthesis has been shown to be safe to use on oral mucosa, this property of material may prevent unexpected tissue reactions.
